# 

**Published:** 1890-12-27

**Authors:** 


### NOTICE TO CORRESPONDENTS.

All MS., Letters, Books for Review, and other matters intended for the Editor should be addressed The Editor, The Lodge, Porchester Square, London, W.

The Editor cannot undertake to return rejected M.S., even when accompanied by stamped directed envelope.

**Notice to Advertisers and Subscribers.**

All Advertisements, Orders for Copies of The Hospital, and Business Communications should be addressed to The Publisher, *not to the Editor*, The Hospital, 140, Strand, W.C.

**To Residents, Secretaries, and Matrons.**

The Editor will be glad to have the Regulations and each Report as issued by Asylums, Hospitals, Convalescent and Nursing Institutions, as well as those of other Charities, and an early copy in duplicate of all Appeals and Festival Notices, etc., addressed to *The Editor, The Lodge, Porchester Square, London, W.* Any superintendent, secretary, matron, or other chief official who regularly sends such information may be registered, on application to the Publisher, to receive free of cost a cover for binding The Hospital in half-yearly volumes.

### BURDETT'S HOSPITAL ANNUAL FOR 1890

May be obtained of the Publisher, The Hospital Offices, 140, Strand, W.C., price 2s. 6d. limp boards, or 3s. 6d. cloth. All orders must be accompanied by a remittance.

### Scraps and Gleanings.

There is an outbreak of diphtheria at Kingston.

Newcastle Hospital for Incurables is to be considerably enlarged.

The Russian Medical Council have prohibited the use of saccharine as an article of food.

Dr. Charles Reid has been elected hon. physician of the Staffordshire General Infirmary.

The Christmas Tree in the children's wards of the London Hospital will be held on December 30th.

Lord Crewe has sent a present of rabbits to the Hospital for Sick Children, Great Ormond Street.

The Italian Minister of the Interior has forbidden the use of Dr. Koch's lymph except in hospitals.

The death has taken place, at Edinburgh, of Mrs. Rutherford, at the great age, it is said, of 101 years.

At Weston super-Mare an entertainment in aid of the Hospital has been held. Nearly £300 was realised.

Mr. W. H. Smith has sent 10 brace of pheasants for the use of the patients at the Grosvenor Hospital.

The Students' Club of Charing Cross Hospital gave a very successful dramatic entertainment to the patients last week.

Lady Zetland has visited the Dublin Royal Hospital for Incurables, and presented Christmas cards to all the inmates.

Two persons suffering from leprosy have been inoculated with the Koch lymph at the Madrid Hospital by their own request.

Mr. Sheriff Harris presided at the annual distribution of prizes to the pupils of the London Society for Teaching the Blind, Swiss Cottage.

Dr. Stainer had an interesting gathering of 400 deaf and dumb children at one of the Board Schools lately. There was tea, conjuring, and marionettes.

Mr. William Beckett, M.P., late of Leeds, by his will empowered his executors to distribute £10,000 to hospitals and other charitable institutions, free of legacy duty.

Wigan has decided to hold Hospital Sunday on January 11th, and Hospital Saturday on February 21st, the proceeds to go to the Royal Albert Edward Infirmary.

Valencia Village Hospital issues its 19th annual report; it is delightful to hear of the good work done in this far-away island off Ireland, by the excellent little hospital.

Sir Edwin Chadwick left a certain sum which will be used to give prizes to sanitary authorities showing the greatest reduction of the death-rate, and to successful teachers of sanitary science.

Stafford Hospital Sunday Committee have met, and have appointed March 8th as Hospital Sunday for next year. This early action has been taken in hope of wider support from the churches and chapels.

Sympathy for the letter carriers in their busy season at Christmas should not be shown by offering them "something to drink." This is the burthen of the annual warning of the Postmaster-General just renewed.

Another death has occurred in Berlin from injection with Dr. Koch's lymph, in the case of a patient who was under treatment by Dr. Libbertz. It is said that Dr. Koch himself admits that in this case the injection was the cause of death.

The Surrey Association for the General Welfare of the Blind, Pelican Buildings, Peckham, London, S.E., has received from Messrs. N. M. Rothschild and Sons a donation of £100 towards the fund being raised for the purchase of their existing premises and workshops.

The Imperial authorities of Russia have invited Sir Joseph Lister, Dr. Koch, and Professor Pasteur to a conference, with a view to founding and establishing a bacteriological institute at St. Petersburg. Sir Joseph Lister was unable to accept the invitation, and Mr. Watson Cheyne left on Tuesday to take his place as the English representative.

The Empress Frederick has sent two glass tubes with Koch's lymph to the Lina Hospital in Naples, accompanied by a long letter to the foundress, the Duchess Ravaschieri. The Empress visited the hospital last year, and takes a great interest in the little patients. It is a children's hospital.

The British Home for Incurables at Clapham Rise is anxious to obtain the services of ladies as Honorary Associates of the charity in town and country, who, when required, would carefully examine into and report upon the circumstances of applicants to be placed on the list of candidates for admission.

Signor Crispi has received the representative of an English syndicate formed for the purpose of transforming Frascati, the ancient Tusculum of Cicero, into an international health and pleasure resort. The Premier assured the representative that the new society would have the support of the Government.

The Society for Promoting Christian Knowledge are again this year distributing a large number of picture cards, small books, &c., among hospitals and similar institutions serving for large centres of population, which have a considerable number of sick children among their inmates, for presentation to these little sufferers on Christmas Day.

The return of Metropolitan pauperism for the first week of December states that the total number of paupers was 94,352, including 59,02[illegible] indoor, and 35,330 outdoor. The numbers in the corresponding period of 1889, 1888, and 1887, were 97,492, 99,831, and 102,559 respectively. The number of vagrants relieved was 913, including 732 men, 164 women, and 17 children.

The Fishmongers' Company seized last month, at Billingsgate, a unfit for human food, 86 tons 13 cwt. of fish. At Shadwell market, out of a total delivery of 1,130 tons, 25 tons were seized, 3 tons being immature fish, and 22 tons Norway herrings. The proportion of fish seized to that delivered was 1 ton in 144 tons at Billingsgate, and 1 ton in 4 tons at Shadwell.

### The Student of Medicine.

[All communications for this Supplement should be addressed to The Editor of The Hospital, at 140, Strand, with the words, "Students' Column," written in the left hand top corner of the envelope.]

### PROFESSOR HUMPHRY ON THE HUMAN FORM.

A lecture on "The Human Form, and its Relation to that of some of the Lower Animals," was delivered on Monday, December 8th, by Professor Humphry, in the Alexandra Hall, Cambridge; Dr. Kenny in the chair. Professor Humphry, after pointing out man's great advantage over all other animals, in his upright position, proceeded to deal with points of difference with regard to the head. One of the special features of man was the great size of his brain in proportion to his body. The brain of man had overgrown the face part of the head, as well as the part behind, and bulged out in all directions; the brain had squeezed all the other parts out of its way. In the lower animals the mouth and nose projected in front; whilst in man, the face was pushed backwards under the skull, the nose pointing downwards, instead of forwards, as in other forms. The teeth being pressed backwards brought the chin into prominence. Other animals did not possess chins, or the "lips of man," which moved in such a delicate and free manner, required for the pronunciation of words. The chin gave to man's lips a facility, freedom and variety of movement not possessed by other animals. In speaking of the teeth, the lecturer said he thought that in the coming race of man, wisdom teeth would not exist. The pressing backwards of the face carried the nose well back between the eyes, so that the two eyes could converge upon one object, so that man thus obtained a greater accuracy of vision than any other animal. The expression and character of man's eyes were due to the same cause. Comparing the skull of the European with that of the Negro, there was more animality about the latter. Was the European's skull developed from the Negro's? Was the Negro's developed from that of the lower animals? This, Dr. Humphry said, was a very large question, into which he was not going to enter; but although observation and reason did tend in the direction of the supposition that man had been developed in some way from some lower animals, it was wonderfully hard to prove. Every bone of man was distinct and recognisable from the bones of other animals, no matter under what circumstances and conditions a bone of man was found, there was never any difficulty in recognising it. The next animal below man did not enter our minds; it was, indeed, remarkable that no nearer approach had been made, in fact, to what seemed so probable in theory.

### LEADING ATHLETES.

Mr. Goodhue, the well-known football player, was born in London, Ontario, Canada, in 1867. In September, 1880, he crossed the Atlantic, and proceeded to the Merchiston Castle School, Edinburgh. The head master at the time was Mr. John J. Rogerson, who was very keen on athletics, and did all he could to encourage them among the boys. Mr. Goodhue remained in the school for five years, and was prominent throughout in the athletic line. During his first three years he worked his way up through the lower XV.'s and XI.'s, playing at almost every position on the field in turn. During the season 1883-4 he played for the first fifteen, and got his cap, Merchiston being the champion Scotch school for the year. In the same year he got into the first eleven, but only had an average of 10. He also won the high jump at the Inter-scholastic Sports (open to all the Scotch schools), clearing 5 ft. 1½ in., but only obtained an equal first in his own school sports. In 1884-85 he again played for the first fifteen, both at forward and also at three-quarters. Also for the eleven, having the top average of 20·9. This year he did not compete at the Inter-scholastic Sports, but won the school high jump, with 5 ft. 7 in., which feat beat the school record by an inch, and was, we believe, the school jump record for the year. At the same time he won the steeplechase and the doubles in the school tennis tournament. In October, 1885, he entered Caius College, Cambridge, and succeeded in getting into the 'Varsity Rugby team in his first year, (playing against Oxford (Cambridge winning by two tries to nil). He played for his College XV. and XI., and won the high jump at the College sports, and the doubles in the College tennis tournament, also rowing No. 5 in the third boat in the Lent races. In 1886-87 he again played for the Varsity XV., which again beat Oxford. In the same year he won the high jump at the College Sports, together with playing for the College XI. and rowing No. 5 in the 1st Lent boat. Early in the following year he having received an injury to his leg was prevented from going in for athletics during the whole year. In October, 1888, he entered at St. Thomas's Hospital, played for the first fifteen, and also joined the London Scottish, for whom he played occasionally. This year Thomas's won the Inter-hospital Cup, and he got his cap at the end of the season. He played for the South v. North at Bradford in February, 1889, the North winning by three goals to nil. He also won the high jump at the Hospital Sports. In 1888-89 he was elected secretary of St. Thomas's Football Club, but the captain having injured his knee was prevented from playing, and so Mr. Goodhue was afterwards elected captain, and Thomas's again won the Inter-hospital Cup for the third year in succession (record). During the same season he played in the following matches: For Middlesex v. Kent, London v. Midland Counties, London and Midland v. Western Counties, London, Midlands, and Kent v. Oxford and Cambridge, Middlesex v. Midlands, Scotland v. Wales, Scotland v. Ireland, and Scotland v. England. During this year he also won the high jump at the Hospital sports, and was second in the United Hospital sports. In 1889-90 he was again elected captain of the hospital fifteen, and also was elected captain of the United Hospital fifteen. He has played twice for Middlesex v. Kent and Surrey; but having played for Scotland, he is now disqualified for all English Rugby Union matches. In addition to all these glories in the athletic world, Mr. Goodhue obtained Honours in Natural Science for his B.A. degree while at Cambridge.

LEADING ATHLETES.

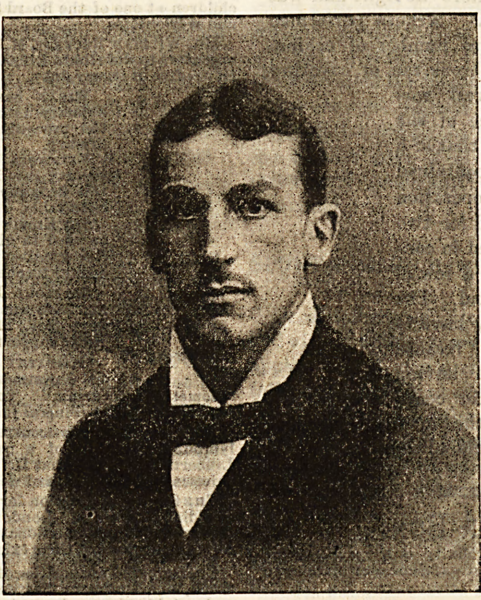

MR. FREDERICK W. J. GOODHUE (St. Thomas's).

### APPOINTMENTS.

At the London Hospital E. C. Williams, B.A., M.B., B.C., Cantab., has been appointed receiving-room officer. At the West London Hospital, Hammersmith Road, F. Herbert Alderson, M.B. Durh., L.R.C.P. Lond., M.R.C.S. Eng., has been appointed house physician, and Balfour Neill, M.R.C.S., L.R.C.P., has been appointed house surgeon. At the South-Western Fever Hospital of the Metropolitan Asylums Board, Albert James Adkins, M.R.C.S., L.R.C.S. Lond., has been appointed clinical assistant. At the County Asylum, Burntwood, near Lichfield, A. C. Farquharson, M.B., C.M. Glas., D.P.H. Camb., F.C.S. Lond., has been appointed senior assistant medical officer. At the St. George's Infirmary, Fulham Road, Joachim Guinane, M.B. Toronto, M.R.C.S. Eng., L.R.P. Lond., has been appointed second assistant medical officer. At the Bolton Infirmary and Dispensary Nathan Raw, M.B., B.Sc., has been appointed senior house surgeon. At the Staffordshire General Infirmary, Stafford, Herbert Shipton, M.R.C.S. Eng., L.R.C.P. Lond., has been appointed house surgeon. At the Glasgow Maternity Hospital H. St. Clair Gray, M.D., C.M. Glas., has been appointed assistant obstetric physician. At the Aberdeen City Hospital W. L. Mackenzie, M.B., C.M. Aberd., has been temporarily appointed assistant medical officer.

### STUDENTS MEDICAL SOCIETIES.

### King's College.

The inaugural address was delivered before the Medical Society of King's College, December 4th, 1890, by Professor Rose, Professor W. R. Smith, president, in the chair, on "A few introductory remarks, followed by a short description of a new modification of the Operation of Inguinal or Iliac Colotomy." Mr. Rose, in

### Students' Medical Societies—(*continued*).

commencing, said that on account of the heavy demands that are at present on his time, his first impulse on receiving the invitation to address this meeting was to decline the honour. On further consideration, however, he felt it his duty to accept the invitation, and to do anything he could for the welfare of his Alma Mater and all connected therewith. In thinking what would be best to bring before the notice of the meeting, the speaker's thoughts strayed back to the time when first he became a member of the society. The meetings in those days were well attended, and the subjects discussed, whether clinical or theoretical, created the keenest interest, and frequently awakened the warmest controversy, so much so that occasions were known upon which one of the members felt it wise to retire from the heat of the discussion to cooler regions, otherwise scenes of an unparliamentary character might have ensued. The chief objects of the society were then touched upon. Professor Rose laid great stress upon the advantages gained by the younger members in writing papers and in entering into the subsequent discussion upon those papers, the paper helping to arrange their ideas in a concise and logical form, the discussion in overcoming nervousness and self-consciousness, and often throwing new light upon certain points in the matter discussed which had hitherto been overlooked. Professor Rose, after kindly criticising the mechanism of the society and offering still more kindly and acceptable advice for its future mangement, referred to the brilliant prospects which the medical school and hospital now possessed. Before concluding these introductory observations the speaker alluded to the loss which Leeds and King's College have recently sustained in the death of Arthur Fergusson MacGill, who was always a keen supporter as a student of King's College Medical Society.

(*To be continued.*)

### Westminster Hospital.

The monthly meeting of the Guthrie Society was held on Thursday, December 11th, at eight o'clock. Mr. C. Stonham, the president, was in the chair, and there were thirty-one members present. Mr. A. Harrison, junior house surgeon, read a paper on three cases of fractured skull, and this was followed by a paper by Mr. Glassington, on "The Relation of Dentistry to Surgery." He divided his discourse into three heads: (1) The Medical Student and Dentistry. The lecturer remarked on the frequency with which general practitioners were met with whose knowledge of the administration of nitrous oxide was of the most meagre description. He also gave it as his opinion that every medical student should be obliged to spend at least three months in the dental department of his hospital, in order to learn how to extract teeth, and what teeth could be preserved. (2) The Dental Student and General Medical and Surgical Knowledge. Mr. Glassington dealt with the quantity of useless lectures, as far as practice was concerned, which a man going in for the L.D.S. only was obliged to attend, such as those on acute specific diseases, &c. (3) The Limits of Dental Surgery. Under this head the author discussed at some length the question whether a man with only a dental qualification should performs operation about the mouth, such as removal of the tonsils, &c., without calling in a regular surgeon, and gave it as a broad rule that he should be allowed to do so in all cases where it was possible to do them in his own consulting room. There was considerable applause at the conclusion of the paper, it having been of a rather lively character, and was followed by a discussion by a large number of students, who gave it as their opinion that they considered themselves quite competent to give gas and extract teeth, and loudly denounced any attempt to burden them with three months' compulsory attendance in the dental department. Some rather caustic strictures were also made on the third section of the paper. Mr. Stonham then read a letter from Mr. Morton Smale, in which opinions rather the opposite to those of the paper were expressed. Mr. Glassington having replied, the discussion closed. There was an exhibition by Mr. Wright afterwards of some of the latest forms of instruments. The meeting terminated with a vote of thanks to Mr. Montague, who had come to explain them.

### NOTES FROM THE SCHOOLS.

### CAMBRIDGE UNIVERSITY.

At Jesus College G. L. Greig, Tonbridge School, has been elected to a scholarship for Natural Science, of £50. At King's College F. B. Stead, Clifton College, has been elected to a scholarship of £50 for Natural Science.

### EDINBURGH UNIVERSITY.

The Edinburgh University Athletic Club gave its annual ball in the Union on the 16th.

Classes meet for the last time before Christmas on the 19th, and re-open on January 6th.

The Hospital Advertiser.

### THE STUDENTS' BOOKSHELF.

We have received a "Manual of Urine Testing," by Mr. John Scott, which includes the physical characters and qualitative and quantitative examination of the urine, together with the clinical information to be derived therefrom. In the preface we are told that the object of the work is to furnish the student and busy practitioner with a handy and reliable guide to the very important branch of clinical study, viz., the examination of the urine. The subject is dealt with in a very useful, practical, and condensed way. Nearly all the appearances, both macroscopical and microscopical, of normal and abnormal urine are tersely tabulated in some twenty small pages, the book being of a convenient size for the waistcoat pocket. Stress is laid on the following excellent rules: (1) When possible, always use two or more tests for any substance. (2) Let your diagnosis be the result of several and successive examinations of the urine. Not only does Mr. Scott give the more ordinary and generally recognised methods for urine testing, but he also gives some more recent and not so generally known ideas—for instance, some special reactions in the case of typhoid urine. In most cases a great number of reactions and tests are given for the various substances found in the urine. At the end of the book are given the rules and directions for the quantitative examination for urea, albumen, and sugar, together with diagrams of the apparatus necessary. The only point in the subject which strikes us as omitted is a description of the various micro-organisms which may be present in the urine, otherwise the manual appears a very complete one of the subject, and well deserving of a place among the small books for the use of students. It is, indeed, a *multum in parvo.*

### PASS LISTS.

### Cambridge, Dec. 16.

Second Examination for Medical and Surgical Degrees.
Michaelmas Term, 1890.

Part I—Pharmaceutical Chemistry.—Examined and Approved: Allan, Cai.; Bagshawe, Cai.; Bolus, B.A., Jesus; A. V. Clarke, Cai.; C. E. Cooper, B.A., Cai.; Cooper, Emman.; Cornaby, non-collegiate; Cowan, King's; Cuff, Joh.; Davies, non-collegiate; Duckworth, Jesus; Garrard, Clare; Gordon, King's; Haigh, Joh.; Hayward, Cai.; A. Hill, B.A., Joh.; Hobday, Christ's; Irving, Cai.; Kent, B.A., Trin.; R. L. Kingsford, Joh.; F. H. Lewis, Joh.; G. P. Mathew, Trin. H.; Maxwell, Corpus; May, Clare; H. J. May, B.A., Cai.; Michael, H. Selw.; Milsome, Trin.; Pead, B.A., Down; Reece, Down; W. A. L. Smith, B.A., Trin.; Thomas, Christ's, Thorman, B.A., Cai.; Tod, Trin.; Trouncer, B.A., Jesus; Twigg, Christ's; Tyson, Cai; C. H. C. Vintner, Cai.; Waldon, Joh.; White, B.A., Sid.; Wicks, B.A., Cai.; Wingfield, M.A., Cai.

Third Examination for Medical and Surgical Degrees.
Michaelmas Term, 1890.

Part 1.—Examined and approved: Abram, B.A., Cai.; Addison, B.A., Cai.; Anderson, B.A., Cai.; Blindloss, B.A., Joh.; G. Calthrop, B.A., Cai.; Carling, B.A., Joh.; Carling, non-collegiate: Craig, B.A., Cai.; Crosby, B.A., Cai; Crump, B.A., Emman.; Dorman, B.A., Clare; Dumbleton, M.A., Pet.; Eccles, B.A., Down.; Felce, B.A., Jesus; A. H. Fisher, B.A., Cai.; Fisher, B.A., H. Cav.; Fooks, B.A., Jesus; A. H. Godson, B.A., Joh.; Gornall, B.A., Cath.; W. Hawker, Trin. H.; Haydon, B.A., Cai.; Horne B.A. Clare; Hulbert, B.A., Trin; Kellock, B.A., Emman.; Latter, B.A., Pemb.; R. Langdon-Down, B.A., Trin.; S. Lewis, B.A., Joh.; Pridham, B.A., Cai.; Roberts, B.A., H. Cav.; Rowland, B.A., Cai.; Samways, M.A., Joh; Savory, B.A., H. Cav.; Shaw, Jesus; Surridge, B.A., Cai.; A. H. Thompson, M.A., Trin.; Turner, B.A., Trin.; G. Wilkinson (jun.), B.A., Emman.; Wood, Christ's; Wrench, H. Cav.

Third Examination for Medical and Surgical Degrees,
Michaelmas Term 1890.

Part II.—Examined and Approved: Batten, B.A., Trin.; Belben, B.A., Christ's; Blaker, B.A., H. Cav.; Buss, Sid.; Conolly, M.A., Cai.; Cordeux, B.A., Cai.; Fosbery B.A., non-collegiate; Gillett, B.A., Sid.; Grabham, B.A., Joh.; Greaves, B.A., Cai.; W. S. Griffith, M.A., Trin. H.; Hale, B.A., King's; Hardwicke, B.A., non-collegiate; S. N. Harrison, B.A., Trin.; Haydon, B.A., Cai.; Kellett, B.A., Joh.; Kellock, B.A., Emman.; Knox, B.A., H. Cav.; C. H. Leaf, B.A., Trin; Martley, B.A., Trin.; Metcalfe, B.A., Trin; Miller, B.A., Queen's; E. J. D. Mitchell, B.A., Cai.; Molson, Emman.; Molteno, B.A., Clare; Newman, B.A., Cai.; Panting, M.A., King's; E. S. Pock, B.A., Christ's; Pinder, Queen's; Pope, B.A., Cav.; Rolfe, B.A., Clare; Samways, M.A., Joh.; W. W. Simmons, B.A., Joh.; H. E. Smith, B.A., Joh.; Sutton, B.A., Clare; Thomas, B.A., Jesus; Usher, B.A., Cai.; Waggett, B.A., Pemb.; Wellford, B.A., Trin.; C. P. White, M.A., Clare; G. Wilkinson, jun., B.A., Emman.; W. Winslow, B.A., Cai.; Wright, B.A., Joh.; Wyman, M.A., Trin.

### ATHLETICS.

### Football.

Association Rules, December 15th.

*Cambridge University* were beaten by *Aston Villa* at Birmingham yesterday by three goals to one.

### Athletics—(*continued*).

RUGBY RULES, December 13th.

*Oxford University v. Edinburgh Academicals.*—This match was played at Raeburn Place, Edinburgh, before four thousand visitors; both teams were well represented. The score on the whole is perfectly indicative of the superiority of Oxford. Their forwards were equal to those of the Academicals, their halves were better, while the three-quarters and back were far superior. In the maul the Dark Blues had the weight, and their speed, tackling, and dribbling came as a surprise to the Scotchmen. Evans, Rice, Patterson, Percival, and the Wilsons were the most prominent. In this game the Oxonian captain was disappointing, well though he had played in England. His kicking and passing was fairly good, but the enthusiasm of his younger schoolmates, who had turned out *en masse*, was not aroused either by his tackling or running. Paul Clauss on the other hand surpassed himself, and was perfect at every point of the game, and in the opinion of many qualified judges he will represent Scotland at Richmond next March. Parkin is a speedy man, and perhaps the best tackler of the three, but he got very few opportunities. P. C. Cochran also distinguished himself. The only feature of the home team was their grand form in the maul. H. J. Stevenson was by far the best three-quarter on the field. The Oxford men kicked off, but M'Ewan and Campbell took the ball to the Oxford line. A lot of kicking went in favour of the Blues, and Clausse and E. S. Wilson took the ball to the centre. At half time neither side had scored. After changing ends, the commencement of the second half was marked by an exhibition of punting by both sides' backs, and here the Scots proved the better. M'Ewan and Glendinning started a dribble, but Parkin kicked the ball dead when dangerous. Now Oxford's passing was seen at its best, and after a good forward rush, Coventry giving Clausse a nice pass, he ran across the line: the place was a difficult one, but Cochran's splendid kick landed a goal. For the next few minutes the game was uninteresting; eventually Fleming had a good low drop, and might have scored, notwithstanding Newton's return, had not Stevenson relieved with a punt into touch. The Blues were pressed by the Academicals, but De Winton and Parkin got the ball away, and the University players at the other end reciprocated. Nothing further was scored, and thus Oxford was victorious in the first match of their tour by one goal to two minors.

*Cambridge University v. Lansdown.*—This match was played at Lansdown Read in delightful weather, in the presence of a large number of visitors. Lansdown played remarkably well, and if they had taken their chance, must have made a draw of the game. The start was made shortly before three o'clock, when Doran kicked off to Wotherspoon, who punted to the half-way flag. From this, Moloney made a good run, and Cambridge was thrown back on their defence. At last the Cantabs came through after some loose scrummaging, Martin Scott picked up the ball and passed it to his brother, he again passed to Hooper, who obtained a try, but the kick failed. In response to M'Gregor's drop Edwards made a magnificent run, but unfortunately passed when near the line; the result was that Rutherford, missing a kick, let Wotherspoon away for some fine passing, the end of which was that Morrison got in this time. Martin Scott kicked the goal, Morrison following his kick up, collared Twigg, which brought about half-time, Cambridge having one goal and one try and Lansdown nil. The first feature of the second half was a good run by Moloney, who, getting in his kick, followed up and nailed M'Gregor, when the whole back division of the enemy got away, pressing up to the very line. Davis brought relief, and gradually Lansdown got up to the Cambridge line, owing chiefly to dribbles by Le Fanu. At last Davis by a foolish pass to Dobbs lost ground, and Hooper, getting well away, collided with Dobbs, who was hurt. This disadvantage was almost immediately cancelled by Nichol twisting his ankle, and Cambridge now got fairly on the run, and with a lot of splendid passing, Hooper, Morrison, and Hooper in this order obtained grand tries; Martin Scott also had an easy shot at goal, and Lansdown had to touch down. Cambridge thus won by two goals and three tries to Lansdown nil.

DECEMBER 16TH.

*Cambridge University* were easily defeated at Bootle by the home club by eight goals to none.

DECEMBER 17TH.

THE LONDON CHALLENGE CUP.—Although there are various games to be played in the first round for this cup, the draw for the second ties has been made as follows: *Chiswick Park* or *Milwall Athletic* v. *Barnes* or *Tottenham Hotspur*, *Old St. Mark's* or *Ilford* v. *St. Bartholomew's Hospital* or *Woodville*, *Clapton* v. *Old St. Stephen's*, *City Ramblers* or *Kildare*, and *Old Westminsters* or *Royal Arsenal* v. *London Caledonians* or *Casuals*.

The *Cambridge University* eleven continued their tour, when they were beaten by the *Blackburn Rovers* by three goals to one at Blackburn.

DECEMBER 15TH.

*Oxford University* were beaten by the *West of Scotland Club*, at Glasgow, by two goals and a try to a try.

*Cambridge University* beat the *North of Ireland* by three goals to one goal and one try, at Belfast.

DECEMBER 16TH.

The *Oxford University* team visited Edinburgh, and gained a victory over *Edinburgh University* by one goal and two tries to nothing.

---

**EVENTS FOR THE MONTH OF DECEMBER.**

**December Meetings and Athletics.**

For list of Students' Medical Societies' Meetings and Football Matches during December, see THE HOSPITAL for Nov. 29th.

### The Hospital Advertiser.

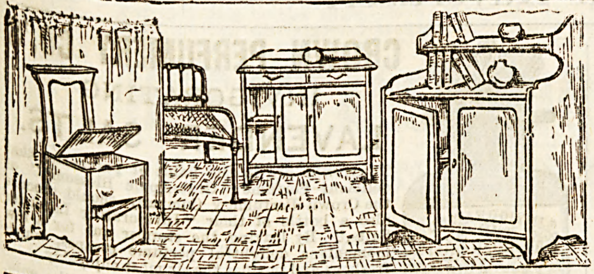

**JENKS & WOOD**

*(Late of Holborn Viaduct),*

**66, BERNERS STREET, LONDON, W.**

**MANUFACTURERS OF**

**Hospital & Sick-room Furniture.**

**HOWE'S PATENT**

**HOSPITAL BED.**

**No. 1.**

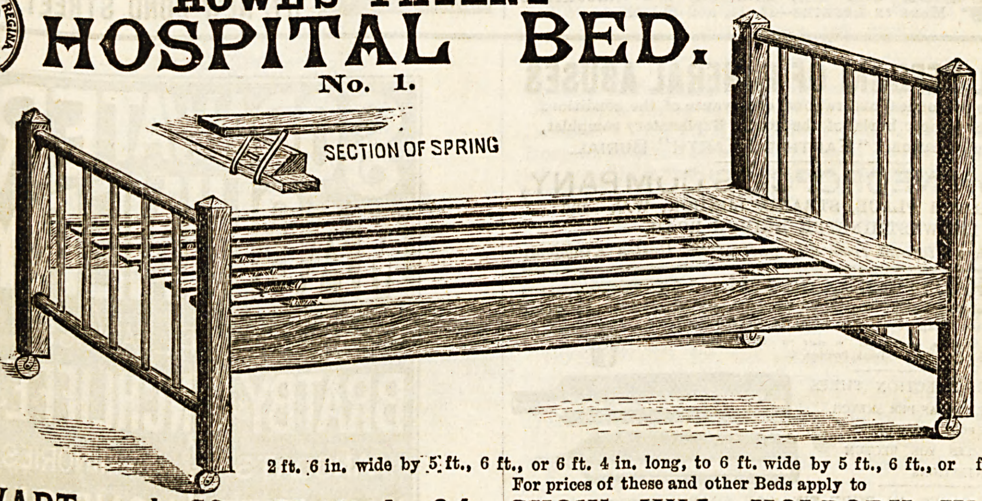

2 ft. 6 in. wide by 5 ft., 6 ft., or 6 ft. 4 in. long, to 6 ft. wide by 5 ft., 6 ft., or ft. in long.

For prices of these and other Beds apply to

**STEWART and CO., Limited, 34, SNOW HILL, HOLBORN VIADUCT.**

ILLUSTRATED PRICE LISTS POST FREE ON APPLICATION. *(See next week's advertisement.)*

### General Charities.

[This Column is devoted to an account of work of charitable institutions other than Medical Charities. The Editor will be glad to receive reports or news of work at 140, Strand. *Philanthropy* should be plainly written on the envelope.]

We have received appeals from the following institutions:—

**THE RAGGED SCHOOL UNION** has now been working among the poor of London for nearly fifty years. We are informed that the general and benevolent funds are overdrawn, and in order to meet the pressing claims of winter, and to continue the varied efforts of the society, help is urgently needed. Secretary, Mr. John Kirk, Exeter Hall, Strand.

**THE CHURCH OF ENGLAND CENTRAL SOCIETY FOR PROVIDING HOMES FOR WAIFS AND STRAYS** ask for special donations to the amount of at least £2,000, in order to enable them to continue their work, and keep out of debt. At the present moment 1,600 children are under the care of the society, which is unable to accept new and deserving cases, and so extend usefully its work. Donations sent to the Church House, Dean's Yard, Westminster, will be gratefully acknowledged by the Secretary, Mr. E. de M. Rudolf.

Owing to heavy expenses in connection with the sanitation and improvement of their schools, the Committee of **THE CLERGY ORPHAN SCHOOLS** have found it necessary to realise a considerable sum by the sale of stock from their capital fund in order to meet a deficit. That they may not have to repeat this, or to diminish the number of children to be elected, increased subscriptions and donations are required, and can be sent to the Secretary, 62, Lincoln's Inn Fields, W.C.

**THE NATIONAL INDUSTRIAL HOME FOR CRIPPLED BOYS**, who through illness or accident have lost a limb or become in other ways disabled, although it has accommodation for one hundred, is unable at the present time to receive more than eighty-five boys, owing to the fact that the Home is encumbered with a mortgage. The pressure of candidates for admission is very great, but till the mortgage is paid off all the available accommodation cannot be made use of. Though the mortgage has been largely reduced, the sum still stands at £2,000. Contributions towards paying off this sum, as well as orders for work in the industrial departments, will be thankfully received by the Secretary, Mr. F. J. Bovis, Woolsthorpe House, Wright's Lane, Kensington, W.

**THE POPLAR MUSICAL UNION** for the musical training and recreation of the Industrial classes, formerly known as the Popular Ballad Concert Committee, appeal for funds to carry on the work of the society. Hon. sec., Mrs. Ernest Hart, 43, Wigmore Street, W.

We regret to hear that the Asylum Chapel of the "little Sons of the Brave," which forms part of the **ROYAL MILITARY ASYLUM, CHELSEA**, is in an unsatisfactory state, and that funds for its restoration are required. Donations should be sent to Colonel Fitzgerald, Royal Miltary Asylum, Chelsea, S.W.

**THE MISSION TO DEEP SEA FISHERMEN**, which has done such good work in the North Sea, asks for immediate financial help in order to enable it to continue to bring relief to the thousands of smacksmen who have so long been neglected. The total contributions for general purposes during the present year have amounted to a little over £15,000, but unless this sum is raised to over £20,000 by the end of the year, the Society will be in debt. Secretary, Mr. Alexander Gordon, 18, Queen Victoria Street, E.C.

Of the 150,000 or more factory girls employed in London alone, many are ill-fed and without any true home. Rough, noisy, and often rude from mere ignorance, because they are brought up amidst sordid surroundings of drunkenness and wretchedness, and with low tastes because they have no refining influences around them, these girls from the very nature of their lives cannot but arouse our sympathy. The factory Helpers' Union, under the auspices of **THE YOUNG WOMEN'S CHRISTIAN ASSOCIATION**, does good work in changing the character and altering the behaviour of many of them, and the Society does an immense amount of good work amongst girls in various employments in London. There are 143 branches, of which 40 are institutes, homes, and restaurants, with a membership of nearly 17,000. The Society now requires £1,000 for present pressing claims, and £21,000 to enable them to purchase institute premises, so as to be rid of heavy rents. Towards this latter sum they have already received nearly £3,000. Secretary, Mr. Henry Kidner, 16A, Old Cavendish Street, W.

